# Supplementary figures and images for: Effect of PM2.5 on burden of mortality from non-communicable diseases in northern Thailand
Source: PeerJ. 2024 Sep 18;12:e18055. doi: 10.7717/peerj.18055 (PMC11416095; doi:10.7717/peerj.18055)

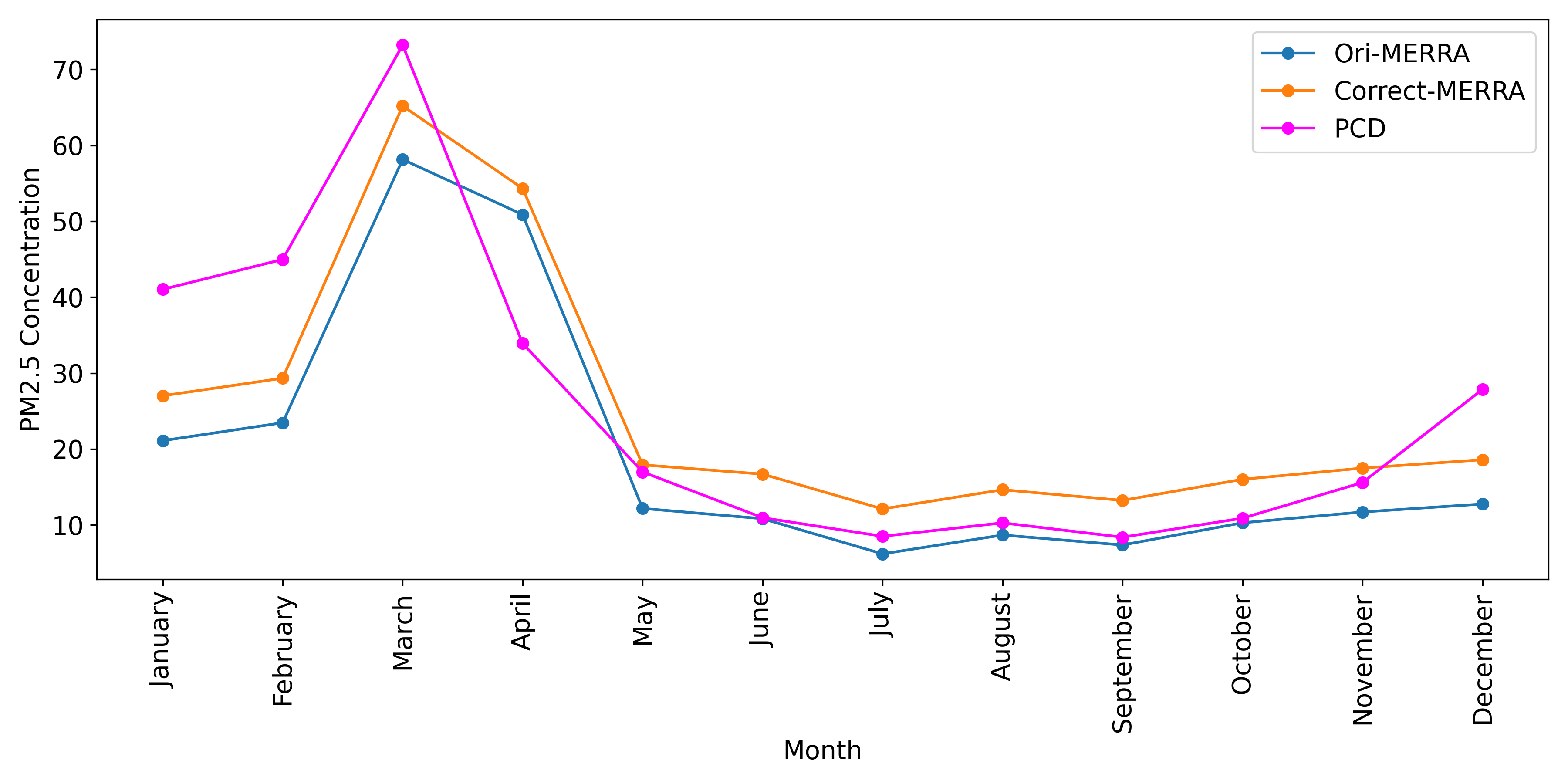

Supplement: Supplemental Information 1 — The plot shows the PM2.5 concentration levels for each month as recorded by the original MERRA-2 (Ori-MERRA), the corrected MERRA-2 (CorrectMERRA), and the Pollution Control Department (PCD) datasets. [file peerj-12-18055-s001.png]

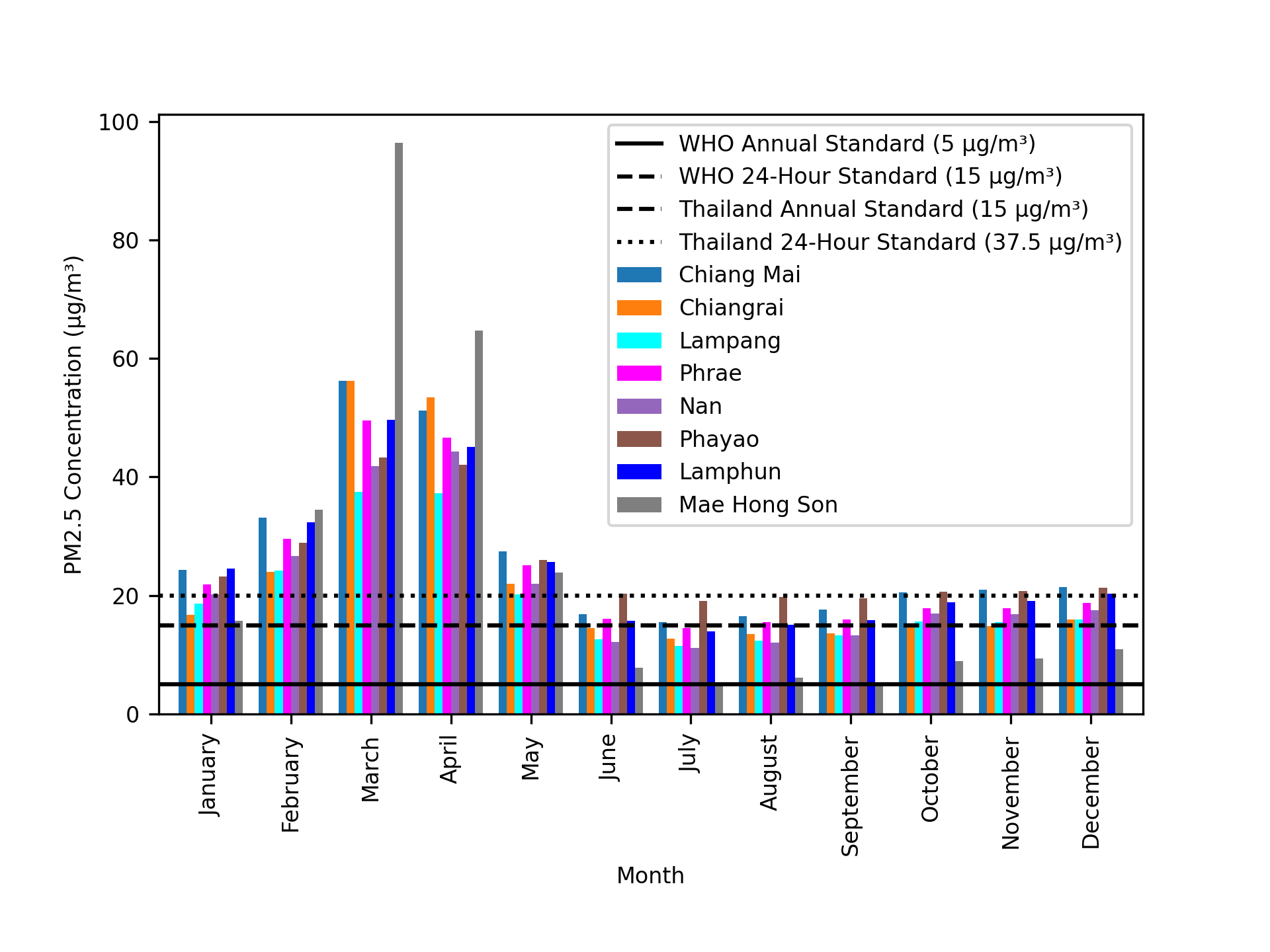

Supplement: Supplemental Information 2 — The bars represent the monthly mean PM2.5 concentrations (µg/m 3) for each province. The horizontal dashed lines indicate the PM2.5 standards: WHO Annual Standard (5 µg/m 3), WHO 24-Hour Standard (15 µg/m 3), Thailand Annual Standard (15 µg/m 3), and Thailand 24-Hour Standard (37.5 µg/m 3). [file peerj-12-18055-s002.png]

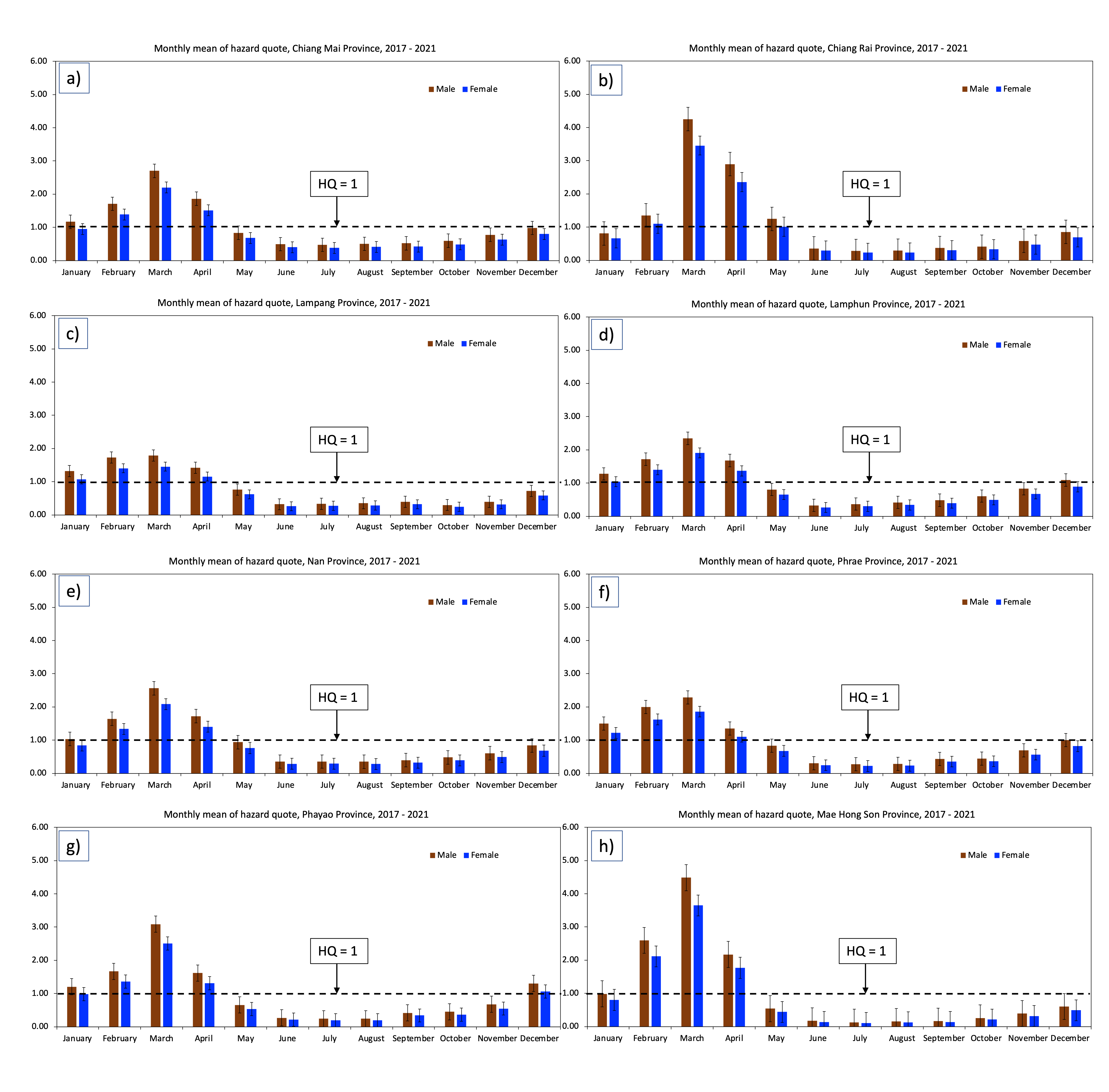

Supplement: Supplemental Information 3 [file peerj-12-18055-s003.png]
