# Supplementary material for: Effect of PM2.5 on burden of mortality from non-communicable diseases in northern Thailand
Source: PeerJ. 2024 Sep 18;12:e18055. doi: 10.7717/peerj.18055 (PMC11416095; doi:10.7717/peerj.18055)
Supplement: Supplemental Information 4 [file peerj-12-18055-s004.docx]

Table 1. Information of seven location from PCD stations in northern Thailand

| No. | Station Name | ID | Location description | Location | |
| --- | --- | --- | --- | --- | --- |
|  |  |  |  | Latitude | Longitude |
| 1 | Chiang Mai Provincial Hall | 35T | Chiang Mai Province | 18.84 | 98.96 |
| 2 | Mae Sai Subdistrict Municipality Office | 73T | Chiang Rai Province | 20.42 | 99.88 |
| 3 | Lampang Meteorological Office | 37T | Lampang Province | 18.27 | 99.50 |
| 4 | Sop Pat Health Promotion Hospital | 38T | Lampang Province | 18.25 | 99.76 |
| 5 | Lamphun Province Stadium | 68T | Lamphun Province | 18.56 | 99.00 |
| 6 | Nan Municipality Office | 67T | Nan Province | 18.78 | 100.77 |
| 7 | Chaloemprakiat Hospital | 75T | Nan Province | 19.57 | 101.08 |
