# Supplementary material for: Effect of PM2.5 on burden of mortality from non-communicable diseases in northern Thailand
Source: PeerJ. 2024 Sep 18;12:e18055. doi: 10.7717/peerj.18055 (PMC11416095; doi:10.7717/peerj.18055)
Supplement: Supplemental Information 5 [file peerj-12-18055-s005.docx]

Table 2. Exposure parameters for uptake through the respiratory system of PM_2.5_.

| Factor | Adult (male) | Adult (Female) | Reference |
| --- | --- | --- | --- |
| Body Weight (BW) (kg) | 67.3 | 57.5 | Liang et al. (2019) |
| Exposure Frequency (EF) (days/year) | 350 | 350 | Morakinyo et al. (2017); Olufemi et al. (2019) |
| Exposure Duration (ED) (years) | 30 | 30 | Morakinyo et al. (2017); Olufemi et al. (2019) |
| Inhalation Rate (IR) (m^3^/day) | 16.6 | 13.5 | Liang et al. (2019) |
| Averaging Time (AT) (days) | 10950 | 10950 | Liang et al. (2019) |
