# Supplementary material for: Effect of PM2.5 on burden of mortality from non-communicable diseases in northern Thailand
Source: PeerJ. 2024 Sep 18;12:e18055. doi: 10.7717/peerj.18055 (PMC11416095; doi:10.7717/peerj.18055)
Supplement: Supplemental Information 6 [file peerj-12-18055-s006.docx]

Table 3 Evaluation Metrics for Correct-MERRA Compared to Ori-MERRA and PCD Datasets

| **Metric** | **Correct-MERRA vs Ori-MERRA** | **Correct MERRA vs PCD** |
| --- | --- | --- |
| MAE | 5.74 | 7.82 |
| RMSE | 5.8 | 9.69 |
| R2 | 0.87 | 0.74 |
